# NC@Bi_2_S_3_ Nanospheres as High-Performance Anode Materials for Lithium-Ion Batteries

**DOI:** 10.1021/acsomega.4c08339

**Published:** 2024-11-26

**Authors:** Wanda Kang, Sen Li, Xingchen Liu, Kun Yan, Wengao Zhang, Youkang Fan, Yuxiang Pan, Jun Feng

**Affiliations:** † Department of Materials Science and Engineering, Southern University of Science and Technology, Shenzhen 518055, Guangdong, China; ‡ Guangdong Provincial Key Laboratory of Functional Oxide Materials and Devices, Southern University of Science and Technology, Shenzhen 518055, Guangdong,China

## Abstract

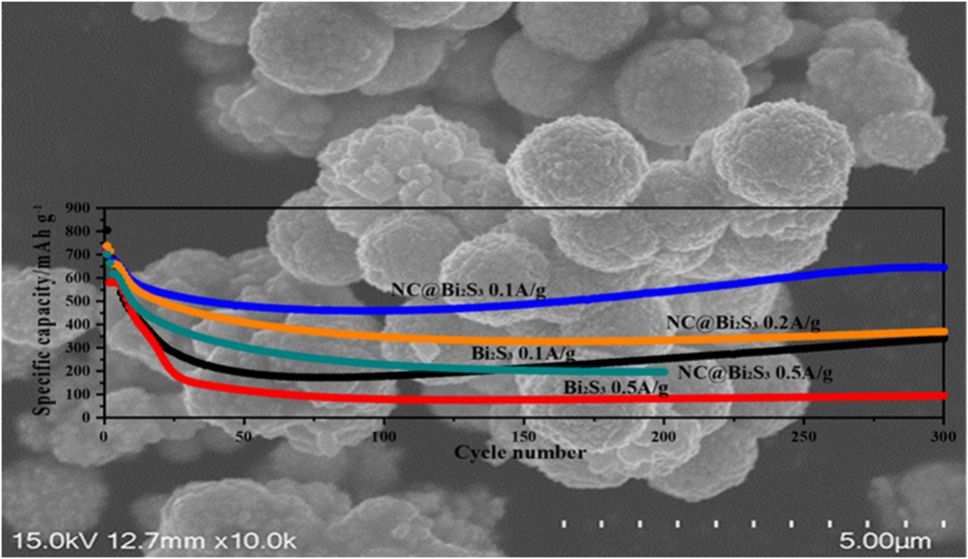

Bi_2_S_3_ holds immense potential to be promoted as an anode material for lithium-ion batteries (LIBs), owing to the high theoretical gravimetric and volumetric capacities. However, the poor electrical conductivity and volume expansion during cycling hinder the practical applications of Bi_2_S_3_. Therefore, through subsequent heat treatment, the nitrogen-doped carbon film was successfully loaded on the nanosphere Bi_2_S_3_, which we call nitrogen-rich carbon layer-coated Bi_2_S_3_ (NC@Bi_2_S_3_). Hence, the nanosphere Bi_2_S_3_ uniformly covered by a nitrogen-rich carbon layer was successfully coated on the Bi_2_S_3_ surface (NC@Bi_2_S_3_) through post-treatment. Due to the effective interaction between glutathione and inorganic materials, dopamine hydrochloride molecules are introduced and polymerized on the surface of the spherical Bi_2_S_3_ structure and then converted into a nitrogen-rich carbon layer with an average thickness of 10.0 nm. The electrochemical tests reveal that the discharge specific capacities of Bi_2_S_3_ and NC@Bi_2_S_3_ reach 340.99 and 645.13 mAh/g after 300 cycles at 100 mA/g, respectively. Kinetic analysis shows that the contribution of pseudocapacitance behavior increases by about 10% after the nitrogen-rich carbon layer is coated. These results suggest the potential of NC@Bi_2_S_3_ as a high-performance anode material for LIBs; the stability can be enhanced by core–shell structures.

## 1 Introduction

With the rapid development of society, the application of new energy as the key technology has gradually grown and developed, including aerospace, military, medical treatment, electric vehicles, etc.^1^ However, new energy sources have the problem of uneven distribution in time and space, and energy storage provides a relatively ideal solution for “peak shaving and valley filling”. Therefore, finding energy storage devices with high energy density is the main trend of future development. Compared with nickel–cadmium batteries, nickel–metal hydride batteries and lead-acid batteries, lithium-ion batteries have the advantages of high energy density, high voltage, low self-discharge, environmental friendliness, and no memory effect, and are considered to be an ideal energy storage solution.^2^ In recent years, the application of lithium-ion batteries has been widely used in people’s daily life. However, commercial anode material graphite shows a limited specific capacity of 372 mAh/g and insufficient rate capability, and there are safety hazards (0.2 V vs Li).^3^ Therefore, it is crucial to find an electrode material with high volumetric specific capacity.

At present, the growth rate of LIBs energy density has stabilized, increasing by less than 3% in the past three decades, failing to meet the growing demand under the current development trend.^4^ Metal oxides and metal sulfides, as active materials for rechargeable lithium-ion batteries, have higher specific capacity and higher operational safety than traditional graphite anodes, and have attracted extensive research.^5^ Among them, metal sulfides, such as iron sulfide, tin sulfide, and bismuth sulfide, have higher charge–discharge specific capacity and better reversible cycle stability than metal oxides. In particular, bismuth sulfide, as a nested indirect bandgap semiconductor with a bandgap of 1.3 eV, has a one-dimensional chain-like crystal structure that can tolerate volume changes during charge and discharge. In addition, although the cost of Bi limits its possibility of replacing low-cost graphite, secondary batteries are widely used, and different applications have different requirements for negative electrode materials. In some scenarios, such as those with high energy density requirements and low requirements for cycle performance (such as drones, military applications, etc.), this type of material still has certain application prospects. Compared with extracting pure metallic Bi from nature, synthesizing Bi_2_S_3_ in the laboratory is more feasible. During the charge and discharge process, it mainly undergoes the conversion reaction of Li^+^ to Bi_2_S_3_. This conversion reaction has good reversibility in Bi_2_S_3_, and the material can restore the original structure during the charge and discharge cycle, maintaining a relatively high capacity and cycle stability. In contrast, MoS_2_ and SnS_2_ usually have a layered structure (two-dimensional structure). Although their layered structure makes it easier to embed/de-embed Li^+^, during the conversion reaction, the layered structure of these materials often undergoes irreversible collapse or phase change, resulting in material failure, and it is difficult to fully restore to the original two-dimensional structure. This irreversible structural change will lead to a decrease in cycle performance. Moreover, Bi_2_S_3_ has a layered structure, provides a safe lithium insertion potential (0.5–0.8 V vs Li) and a mass specific capacity of 625 mAh/g, and is considered to be one of the excellent candidates for lithium-ion batteries.^6^ However, when studying the storage application of bismuth sulfide anode lithium-ion batteries, a series of problems followed, including excessive volume change during the cycle, low electronic conductivity, and the precipitation and dissolution of polysulfide intermediates in the electrolyte during charge and discharge, resulting in low material utilization and rapid capacity decay.^7^ Moreover, the discharge reaction of Bi_2_S_3_ involves a more complex electrochemical process, which usually includes the alloying and conversion reaction of lithium and Bi_2_S_3_, so its discharge voltage is relatively high, usually between 0.5 and 1.0 V. As a negative electrode material, graphite stores lithium ions through lithium insertion reaction. Its chemical reaction is relatively simple, and the discharge voltage is generally low, usually between 0.1 and 0.3 V. The lithium storage mechanism of bismuth sulfide as the anode electrode is completed through conversion and alloy reaction, and its mechanism can be expressed by the following formula:
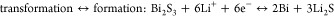
1
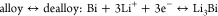
2

In recent years, researchers have studied bismuth sulfide materials to address the above issues, such as encapsulating bismuth sulfide nanorods with carbon nanotubes, designing new daisy-shaped bismuth sulfide nanostructures, and strongly coupling Bi_2_S_3_@CNT hybrids.^8^ Influenced by thistle plants, Liang et al.^9^ designed and synthesized a flower-like morphology of bismuth sulfide, which is composed of nanowires with an average size of 5–10 nm. This flower-like morphology has a large specific surface area and can be exposed to the electrolyte to the greatest extent. It can quickly exchange ions with the electrolyte. At the same time, this structure also has a certain mechanical flexibility, which can alleviate the impact of the volume effect. It shows better cycling performance than nanorods. When it is cycled 100 times at a current density of 300 mA/g, it still has a specific capacity of ∼200 mAh/g. Bai et al.^10^ synthesized nanospherical bismuth sulfide. The cycling performance of bismuth sulfide with this morphology is also unsatisfactory. When charged and discharged at a current density of 200 mA/g, it showed a cycling performance only close to that of nanorods. After 20 cycles, only ∼100 mAh/g of specific capacity remained. Yue et al.^11^ designed and synthesized a bismuth sulfide with a lemongrass-like morphology. This morphology is very similar to the morphology of thistle flowers. The difference is that this morphology is composed of nanorods with a diameter of 100 nm, and there are many mesopores on the surface of the nanorods. This material shows very excellent cycling performance. After 100 cycles at a current density of 200 mA/g, it still has a specific capacity of ∼600 mAh/g. The above research mainly focuses on controlling the micromorphology of Bi_2_S_3_. The problem of poor conductivity of Bi_2_S_3_ cannot be fundamentally improved by changing the micromorphology. However, nitrogen-rich carbon materials can significantly improve conductivity, alleviate volume expansion and stabilize the electrode interface, which leads us to load a layer of nitrogen-rich carbon layer material on the surface of Bi_2_S_3_, and there is less research on the surface coating treatment of Bi_2_S_3_, but this issue is very important for the study of energy storage characteristics of lithium battery anode materials.

To solve the problems of volume effect, shuttle effect, and rapid capacity decay of lithium battery anode materials. In this study, spherical Bi_2_S_3_ was synthesized by a new hydrothermal method, and NC@Bi_2_S_3_ was successfully synthesized by subsequent heat treatment. The electrode microstructure phase was characterized by XRD, TEM, and XPS, and the lithium storage performance was studied by electrochemical testing. The study showed that dopamine hydrochloride was introduced and polymerized on the surface of spherical Bi_2_S_3_, and then converted into a nitrogen-rich carbon layer after mild annealing. Since there are many voids on the surface of Bi_2_S_3_, the coating of the nitrogen-rich carbon layer can effectively fill the voids, buffer the volume change of Bi_2_S_3_ during the cycle process, accelerate the speed of electron conduction, and inhibit the precipitation of polysulfide intermediates during the cycle, thereby improving the adsorption capacity of polysulfide. This article discusses this from a scientific perspective.

## 2 Experiments

Bismuth nitrate pentahydrate (Bi(NO_3_)_3_·5H_2_O), glutathione (GSH), triaminemethane (C_4_H_11_NO_3_), ethanol, and dopamine hydrochloride (C_8_H_12_ClNO_2_) were purchased from Aladdin. All chemicals and reagents are used as received.

### 2.1 Synthesis of Spherical Bi_2_S_3_

Dissolve 0.2425g of anhydrous bismuth nitrate (Bi(NO_3_)_3_·5H_2_O) in 30 mL of deionized water and stir vigorously for 1 h. Then, add 0.3073 g of glutathione (GSH) and continue stirring for an additional hour until a turbid solution is obtained. Transfer the solution into a homogeneous reactor with a polytetrafluoroethylene (PTFE) liner and react at 120 °C for 12 h. After cooling to room temperature, filter the mixture using deionized water and alcohol to isolate a black precipitate. Finally, dry the precipitate in an oven at 60 °C for 12 h to yield spherical Bi_2_S_3_ structures.

### 2.2 Synthesis of NC@Bi_2_S_3_ Composites

The prepared spherical Bi_2_S_3_ (0.306 g) was dispersed in 750 mL of deionized water and stirred for 5 h to form a uniform solution. The pH of the solution was adjusted to approximately 8.5 by gradually adding tris(hydroxymethyl)aminomethane (C_4_H_11_NO_3_) while monitoring the pH meter. Next, 0.034 g of dopamine hydrochloride (C_8_H_12_ClNO_2_) was added, and the mixture was stirred overnight. The resulting solution was filtered using deionized water and alcohol to obtain a black precipitate, which was dried in an oven at 60 °C for 12 h. The dried product was then carbonized in a tubular furnace under an argon atmosphere at 500 °C for 1 h to yield NC@ Bi_2_S_3_.

### 2.3 Polysulfide Adsorption Experiment

The Bi_2_S_3_ and NC@Bi_2_S_3_ lithium-ion half-cells, after cycling for 300 cycles, were disassembled in a glovebox. All components, except the anode material (separator), were immersed in *N*-methylpyrrolidone (NMP) solution and left undisturbed in the glovebox for 3 days.

### 2.4 Characterization

The crystal structure and morphology of the prepared samples were studied by X-ray diffraction analysis (XRD - Smartlab 3KW, Cu Kα radiation λ = 1.54 Å). The morphology and microstructure of the samples were observed by scanning electron microscopy (SEM, SU8200) and transmission electron microscopy (TEM, SU8230), and the composition and elemental valence of the samples were further characterized by X-ray photoelectron spectroscopy (XPS, Escalab Xi^+^).

Nitrogen adsorption–desorption isotherms were measured using a Micromeritics ASAP 2460 analyzer. The average pore size distribution and specific surface area were calculated using the Barrett–Joyner–Halenda (BJH) model and the Brunauer–Emmett–Teller (BET) equation, respectively. Thermogravimetric (TGA) curves were obtained on a NETZSCH-STA-409 CD thermal analyzer, heating from room temperature to 650 °C atmoph at a heating rate of 10 °C min^–1^ under an argon atmosphere.

### 2.5 Electrochemical Testing

The electrochemical performance of Bi_2_S_3_ and NC@ Bi_2_S_3_ in CR-2032 coin cells was studied. The active material, carbon black and binder (carboxymethyl cellulose, CMC) were composed in a weight ratio of 7:2:1. The solvent was deionized water, and the active material slurry was prepared after stirring for 12 h. Then, the mixture was coated on a copper foil current collector and dried in a vacuum oven at 80 °C for 12 h to prepare a working electrode. Lithium metal foil was used as the counter electrode in the half-cell, Whatman GF/A glass fiber filter was used as the separator, the electrolyte was 1.0 mol L^–1^ LiPF_6_, and the solution was ethylene carbonate-dimethyl carbonate (1:1 vol %). All coin cells were assembled in a glovebox. The lithium storage performance of the prepared bismuth sulfide nanomaterials was tested. The constant current charge and discharge processes were carried out in the voltage range of 0.01–3.0 V vs Li/Li^+^ and at different current densities using a LAND CT2001A battery tester system. Cyclic voltammetry (CV) and electrochemical impedance spectroscopy (EIS) were recorded on a CS2350H electrochemical workstation at ambient temperature (∼25 °C).

## 3 Results and Discussion

**Figure 1:**
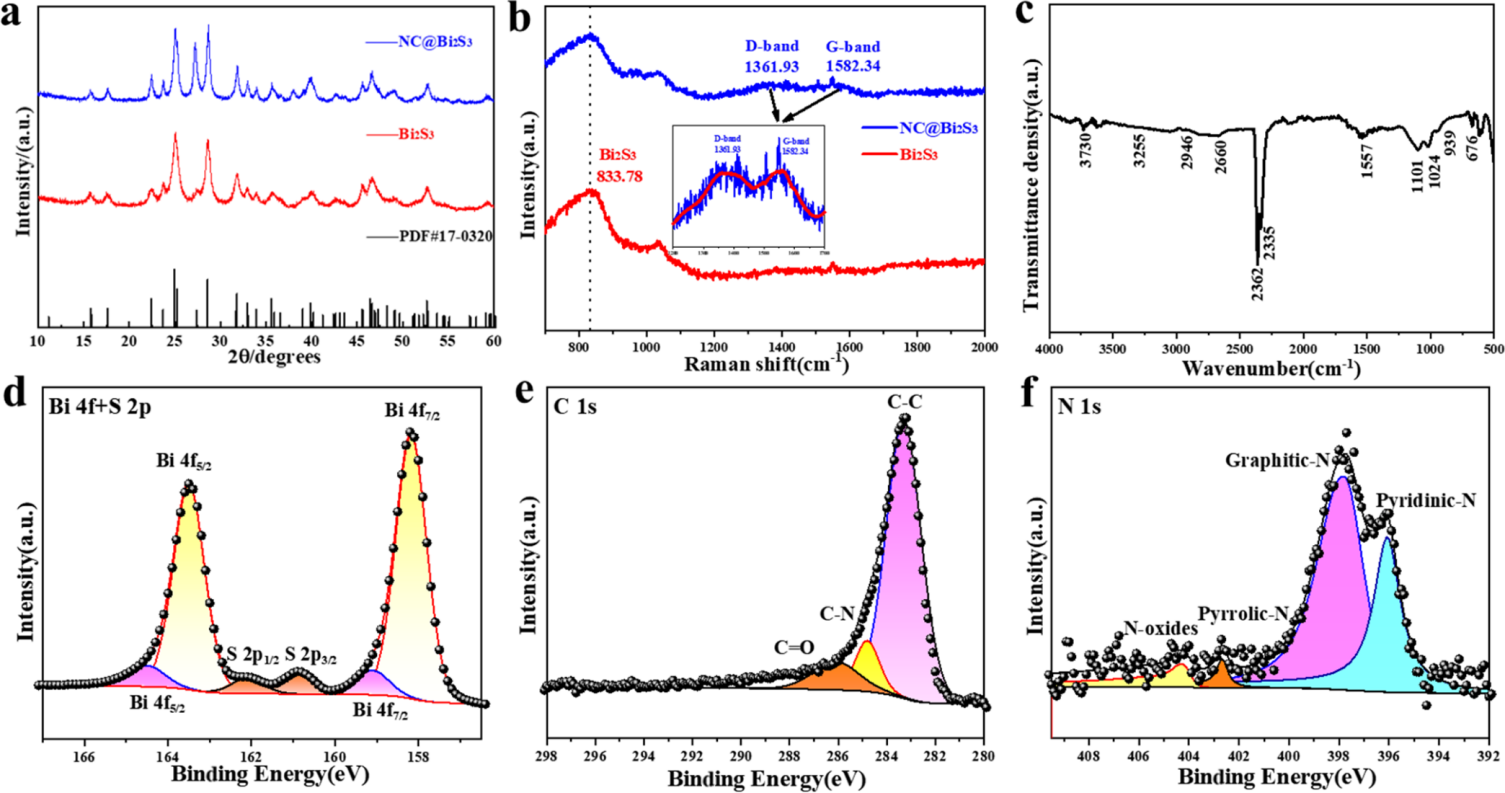
Structural characterization of spherical Bi_2_S_3_ and NC@Bi_2_S_3_ composites (a) XRD patterns of spherical Bi_2_S_3_ and NC@Bi_2_S_3_ composites; (b) Raman spectra of spherical Bi_2_S_3_ and NC@Bi_2_S_3_ composites; (c) FTIR spectra of NC@Bi_2_S_3_ composites; High-resolution XPS spectra of (d) Bi 4f and S 2p; (e) C 1s and (f) N 1s.

The crystal structures of Bi_2_S_3_ and NC@Bi_2_S_3_ were analyzed by X-ray diffractometer. As shown in Figure 1a, both showed standard diffraction peaks, corresponding to standard orthorhombic Bi_2_S_3_ (PDF#17-0320), with lattice parameters of *a* = 1.115 nm, *b* = 1.130 nm, *c* = 0.3981 nm. No diffraction peaks of other impurities were found, indicating that the purity of the two was high. In addition, the diffraction peak intensity of NC@Bi_2_S_3_ at 2θ = 27.476°, corresponding to the (021) crystal face, was significantly enhanced compared with Bi_2_S_3_, which may be due to the improved crystallinity caused by the mild heating treatment during the coating process. No diffraction peaks of the nitrogen-rich carbon layer were observed in the pattern, because carbon is mainly in an amorphous state.^12^ In order to further verify the existence of carbon and study the degree of shown in Figure 1b. The results show that the characteristic band at a wavenumber of 834 cm^–1^ is related to the surface phonon mode of Bi_2_S_3._^13^ The other two strong peaks at 1362 and 1582 cm^–1^ correspond to the D band (defective disordered carbon, E2g) and G band peaks (hybridized graphene carbon, A1g) of carbon materials, respectively. The ratio of the D band and G band intensities (*R* = *I*_D_/*I*_G_) is 1.05, which is slightly higher than that of pure graphene oxide (*R* = 1.02). This result shows that more defects are generated in the process of fully reducing graphene oxide with the assistance of visible light.^14^ The Fourier transform infrared (FTIR) spectrum of the NC@Bi_2_S_3_ composite is shown in Figure 1c. The broad absorption peak at 3255 cm^–1^ is attributed to the O–H group of H_2_O, and the broad absorption peaks at 2946 and 2660 cm^–1^ are attributed to the −CH_3_ and −CH_2_ groups.^48^ The strong peak observed at 1557 cm^–1^ is attributed to the stretching mode of the C–N group, indicating that a nitrogen-rich carbon layer complex nanostructure is formed in the synthesized Bi_2_S_3._^49^ The peaks at 1101, 1024, and 1557 cm^–1^ are due to the symmetric stretching vibrations of the N–O group, C–O bond and CO_2_, respectively.^15^ The peaks at 939 and 676 cm^–1^ can be attributed to the Bi–S bond. Next, the surface state of the NC@Bi_2_S_3_ composite was analyzed by X-ray photoelectron spectroscopy (XPS). The full spectrum is shown in Figure S1. Bi, S, O, N and C elements can be detected, among which the appearance of O element may be caused by the adsorption of air on the sample surface. The high-resolution XPS spectra of Bi 4f and S 2p of the sample are shown in Figure 1d. The Bi 4f_7/2_ and 4f_5/2_ peaks are located at around 158.2 and 163.5 eV, respectively. There are also two weak peaks at 160.9 and 162.1 eV, which belong to S^2–^ substances S 2p_3/2_ and S 2p_1/2_, respectively. The presence of these elements indicates that sulfur atoms are combined with bismuth. Figure 1e shows the presence of C–C (283.30 eV), C–N (284.87 eV) and C=O (287.20 eV).^16^ Among them, C–C and C–N may come from the decomposition of dopamine hydrochloride. The high-resolution XPS spectrum of N 1s is shown in Figure 1f. The N 1s peak consists of two main peaks. The peak at 395.6 eV belongs to pyridinic nitrogen, while the peak at 397.5 eV belongs to graphitic nitrogen, and the peaks at 402.8 and 404.3 eV belong to pyrrolic nitrogen and N-oxide, respectively. The presence of four nitrogen atoms indicates that N atoms are successfully doped into the amorphous carbon layer during the carbonization process.^17^ The atomic percentage of each element obtained in the XPS analysis before and after coating is shown in Table 1. After coating, the atomic percentage of C increased by 37.77% (significant increase), and the atomic percentage of N increased by 1.36% (weak increase); The atomic percentage decreased by 5.14%, and the atomic percentage of S decreased by 32.21%. The increase in carbon and nitrogen content indicates that the nitrogen-rich carbon layer was successfully formed. The decrease in Bi and S may be due to the material being partially covered or reacting during the coating process, resulting in a decrease in surface elements. The above results show that the types of nitrogen are mainly highly active pyridinic nitrogen and N-oxide with high electronic conductivity. The abundant nitrogen elements in the amorphous carbon layer provide more lithium reaction sites and improve the adsorption capacity of polysulfides due to the increase in polarity, which plays an important role in improving electrode kinetics and maintaining structural integrity. According to the thermogravimetric analysis (TGA) in Figure S2, the weight percentage of carbon in the composite material was determined to be 9 wt %.

**Table 1:** Atomic Percentage of Each Element in the XPS Full Spectrum Analysis of Bi_2_S_3_ and NC@Bi_2_S_3_

	**XPS (atom %)**				
**sample**	**Bi**	**S**	**C**	**N**	**O**
Bi_2_S_3_	7.69	52.06	25.25	3.62	11.39
NC@Bi_2_S_3_	2.55	19.85	63.02	4.98	9.60

**Figure 2:**
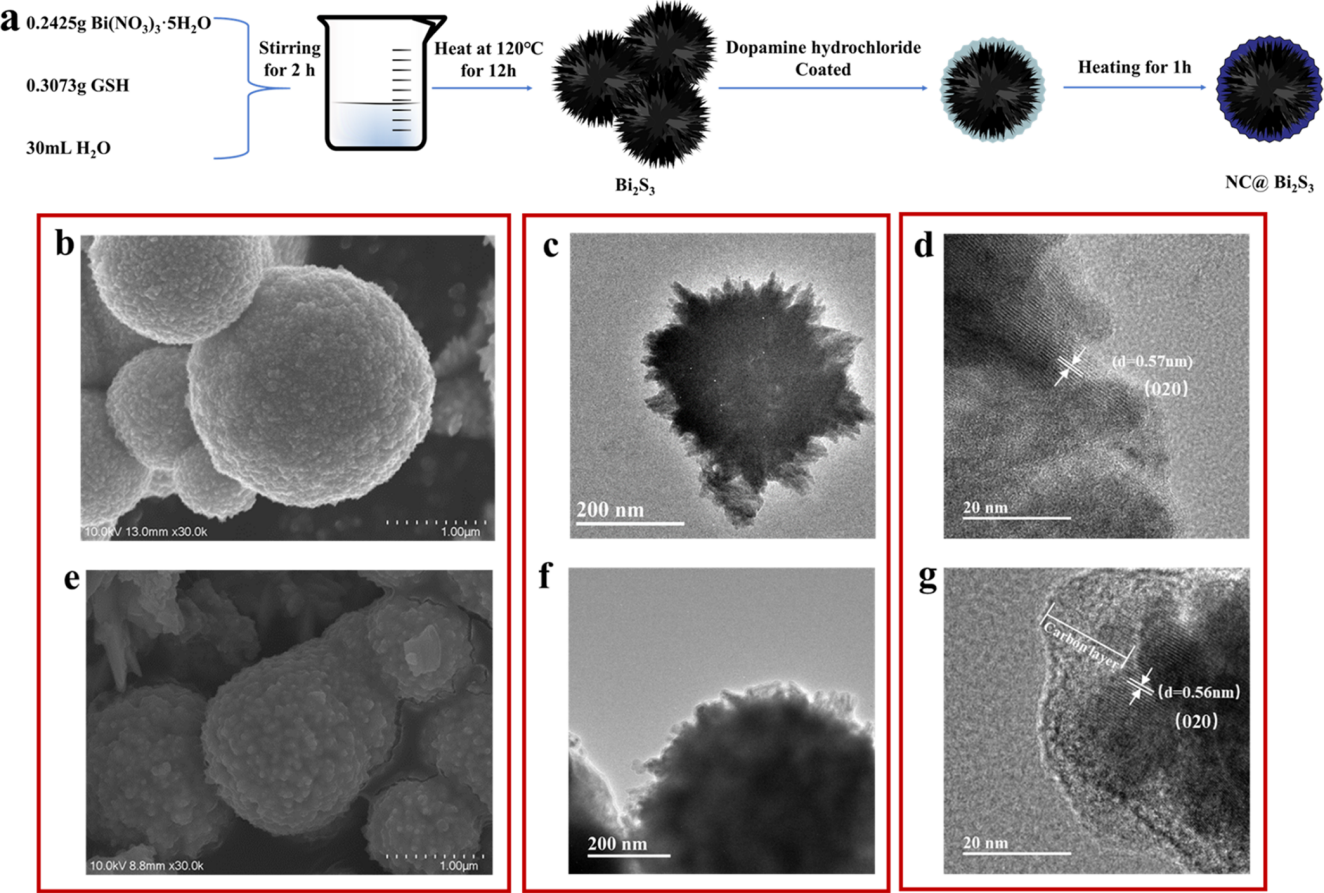
(a) Schematic diagram of the formation of spherical NC@Bi_2_S_3_ structure; (b) SEM image of the original spherical Bi_2_S_3_; (c and d) TEM images of the original spherical Bi_2_S_3_; (e) SEM image of NC@ Bi_2_S_3_ structure; (f and g) TEM images of NC@ Bi_2_S_3_ structure.

The formation diagram of NC@Bi_2_S_3_ structure is shown in Figure 2a. Bi(NO_3_)_3_·5H_2_O and GSH act as providers of bismuth and sulfur, respectively. During the reaction, S^2–^ released by GSH combines with Bi^3+^ released by Bi(NO_3_)_3_·5H_2_O to generate Bi_2_S_3_. Subsequently, triaminemethane is added to neutralize the acidic substances in the solution to regulate the electrostatic interaction between colloidal nanorods.^18^ After dopamine hydrochloride molecules are introduced and polymerized onto the surface of spherical Bi_2_S_3_, Bi_2_S_3_ coated with a nitrogen-rich carbon layer is obtained after annealing. The image of uncoated Bi_2_S_3_ observed under a SEM is shown in Figure 2b. The morphology of the precursor can be clearly observed, and the diameter is in the range of 1–2 μm. The high-resolution surface image is shown in Figure S3. It can be observed that the particles on the surface of the sphere form a columnar cross structure, and there are obvious depressions. Compared with the precursor Bi_2_S_3_, as shown in Figure 2e, NC@Bi_2_S_3_ shows the same microspherical morphology, but the surface of the sphere is coated with a nitrogen–carbon layer, and the diameter is in the range of 1–2 μm. (Low-resolution SEM images of Bi_2_S_3_ and NC@Bi_2_S_3_ are shown in Figure S4). It can be inferred that the formation process of spherical Bi_2_S_3_ is generated by the gradual superposition of rods.

The morphology and structure of Bi_2_S_3_ and NC@Bi_2_S_3_ were further studied by TEM and HRTEM. As shown in Figure 2c, it can be clearly seen that there are many gaps on the surface of the precursor. As shown in Figure 2f, It can be observed that the gaps on the surface are filled with nitrogen–carbon layers, which matches the lower specific surface area (6.45m^2^/g, see Figure S5) and shows a uniform profile. In the high-resolution TEM image of Figure 2d, lattice fringes with an interlayer spacing of 0.57 nm can be clearly observed, which corresponds to the (020) crystal plane of Bi_2_S_3_. It can still be found that the surface of the precursor Bi_2_S_3_ is not flat. In the high-resolution TEM image of Figure 2g, it is observed that the surface is covered with a layer of transparent material, confirming the existence of a nitrogen-rich carbon layer with an average thickness of 10 nm. It can also be clearly observed that the lattice fringes with an interlayer spacing of 0.56 nm correspond to the (020) crystal plane of Bi_2_S_3_. The transparent area on the surface of NC@Bi_2_S_3_ explains the existence of amorphous and nitrogen-rich carbon layers.

It is worth noting that the nitrogen-rich carbon layer is in close contact with the surface gap of the spherical Bi_2_S_3_, and there is no obvious gap, which will help improve the transfer efficiency of electrons and Li^+^ at the interface. This uniform and tightly contacted nitrogen-rich carbon layer originates from the strong affinity of dopamine hydrochloride to the substrate, which will help improve the conductivity and structural stability of the NC@Bi_2_S_3_ anode during the charge and discharge process and the lithiation/delithiation process.^19^

**Figure 3:**
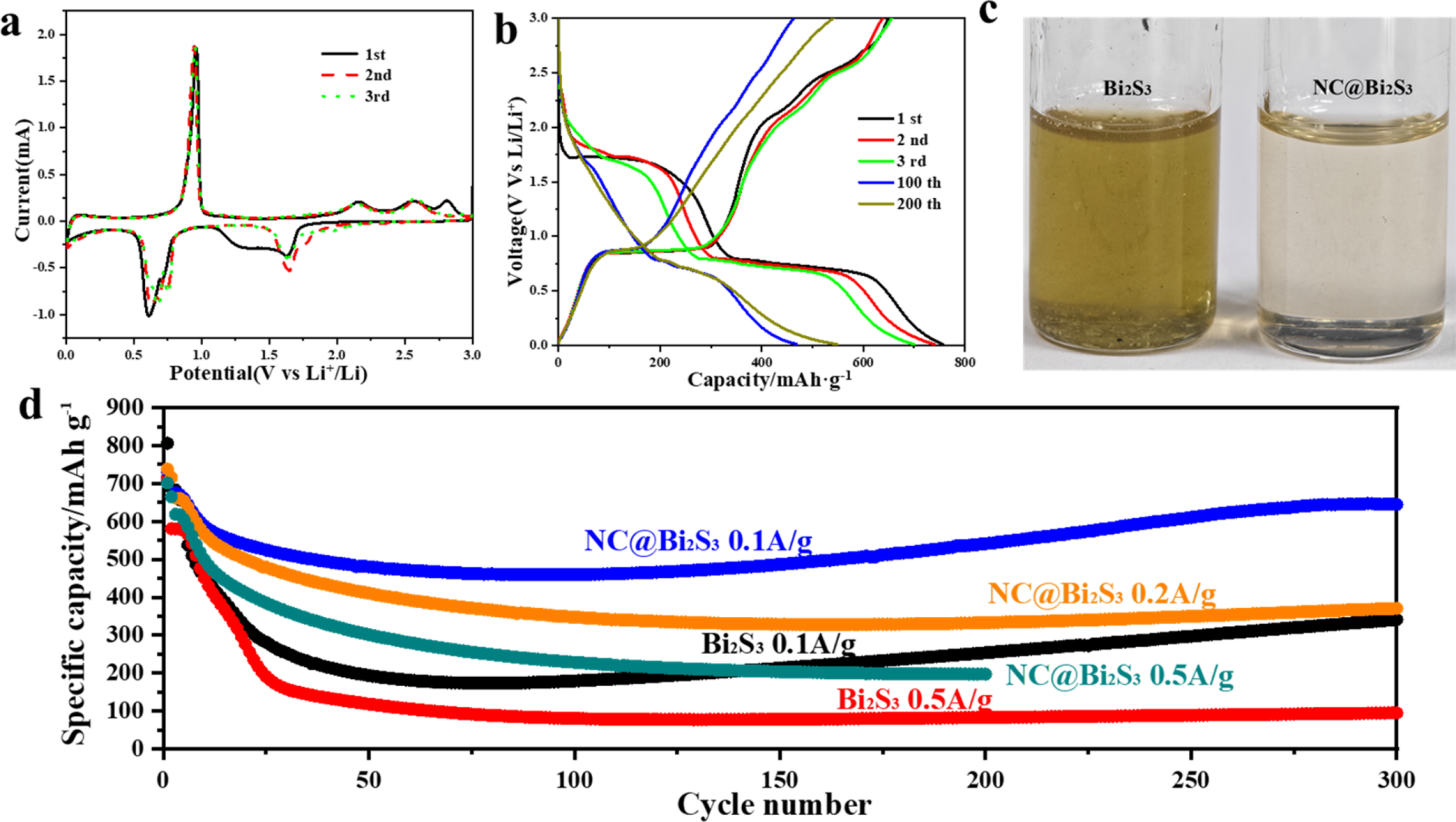
(a) Cyclic voltammetry curves of NC@ Bi_2_S_3_ battery at a scan rate of 0.1 mV/s for the first three charge and discharge cycles; (b) Constant current charge and discharge curves of NC@Bi_2_S_3_ battery at different cycle numbers at a current density of 0.1 A/g; (c) Comparison of S dissolution of Bi_2_S_3_ and NC@Bi_2_S_3_ batteries after 300 cycles; (d) Cyclic performance of Bi_2_S_3_ and NC@Bi_2_S_3_ batteries at different current densities.

The Bi_2_S_3_ battery coated with a nitrogen-rich carbon layer exhibits good electrochemical performance during the cycle process. Figure 3a is the CV diagram of the first three charge and discharge cycles of the NC@Bi_2_S_3_ battery (scan rate 0.1 mV/s). It can be seen that the redox peaks of the first three cycles have a good overlap (Figure S6a shows the CV graph of the first three cycles of bare Bi_2_S_3_), indicating that the irreversible loss of the material in the first cycle is small. During the first discharge, the first reduction peak was observed at 1.64 V, which was caused by the conversion of Bi_2_S_3_ to metallic Bi and Li_2_S. Subsequently, two adjacent reduction peaks were observed at 0.71 and 0.61 V, which were due to the alloying reaction of Bi_2_S_3_, where metallic Bi and Li^+^ formed LiBi and Li_3_Bi alloys. During the first charge, the first oxidation peak was observed at 0.96 V, which was due to the dealloying reaction of Li_3_Bi alloy to reduce to Bi metal. In addition, weak oxidation peaks were also observed at 2.16 and 2.55 V, which may be related to the regeneration of Bi_2_S_3_ and the extraction of lithium ions from layered Bi_2_S_3_, respectively.^20^ In the subsequent two or three cycles, the electrode showed stable and reproducible peak behavior, indicating that the lithium storage process of NC@Bi_2_S_3_ electrode is reversible. It is worth noting that the broad reduction peak at about 1.3 V during the first discharge did not appear in the next two cycles, indicating that the peak may be caused by the formation of solid electrolyte interface (SEI) film. These peaks in the CV curve match the constant current charge–discharge (GCD) characteristic curve, and their voltage platforms correspond to the peaks on the cyclic voltammetry (CV) curve.

Figures 3b and S6b show the constant current charge–discharge curves of NC@Bi_2_S_3_ and Bi_2_S_3_ batteries at different cycle numbers at a current density of 0.1 A/g, respectively. The two pictures show the same charging and discharging platform. During the discharge process, there are two obvious flat platform at 1.71 and 0.75 V, corresponding to the conversion reaction of Bi_2_S_3_ and the alloying reaction of Bi, respectively. In the subsequent charging process, due to the dealloying reaction of LiBi and Li_3_Bi alloy, a flat platform appeared at around 0.88 V, followed by two fuzzy potential platforms at around 2.1 and 2.5 V. The potential platform reflected in the discharge/charge curve corresponds well to the redox peak observed in the CV curve. It is worth noting that the second and third charge–discharge cycle curves coincide well with the curve of the first cycle, indicating that it has good stability during repeated lithiation/delithiation.^21^

At the same time, we tested the ability of the nitrogen-rich carbon layer to inhibit polysulfide dissolution. As shown in Figure 3c, the battery leachate without nitrogen-rich carbon layer coating showed sulfur yellow, while the battery leachate with nitrogen-rich carbon layer coating was transparent and colorless, indicating that the nitrogen-rich carbon layer coating can effectively inhibit polysulfide dissolution, making the battery have good cycle stability. The long-term cycle performance diagram of Bi_2_S_3_ and NC@ Bi_2_S_3_ batteries is shown in Figure 3d. At current densities of 0.1 and 0.2 A/g, the specific capacity of NC@ Bi_2_S_3_ battery decreased slightly in the first 30 cycles and stabilized in the later period. After 300 cycles, the specific capacities reached 645.13 and 370.62 mAh/g, respectively. Compared with the initial specific capacity, the specific capacity retention rate reached 88%. This retention is much better than that of some Bi_2_S_3_ nanostructures, indicating that our nitrogen-rich carbon layer coating process is effective (Table 2). After 200 cycles at a current density of 0.5 A/g, the specific capacity still reached 197.24 mAh/g. In comparison, the Bi_2_S_3_ battery has a specific capacity of only 340.99 and 95.04 mAh/g after 300 cycles at current densities of 0.1 and 0.5 A/g. Compared with pure Bi_2_S_3_, the specific capacity and cycling stability of NC@ Bi_2_S_3_ are significantly enhanced, indicating the importance of the nitrogen-rich carbon layer. The carbon coating aids electron transport across the electrode and increases the efficiency of utilization of the active Bi_2_S_3_ material.^22^

**Table 2:** Comparison of Li Storage Capability of the NC@Bi_2_S_3_ with Other Bi_2_S_3_ and Sulfide Materials

**materials**	a**retention (%)**	**rate capability**	**refs**
Bi_2_S_3_	59% over 10 cycles		ref^43^
Bi_2_S_3_	40% over 50 cycles		ref^44^
Bi_2_S_3_	27% over 30 cycles		ref^45^
Bi_2_S_3_@C	78% over 100 cycles		ref^46^
SnS_2_@CNT	b34% over 50 cycles	296 mAh/g at 0.5 A/g	ref (47)
**NC@Bi**_**2**_**S**_**3**_	c**88% over 300 cycles**	**645.13** mAh/g at 0.1 A/g	**this work**

a Ratio against the theoretical capacity;

b Theoretical capacities of SnS_2_ is1231 mAh/g, respectively;

c The contribution of Bi_2_S_3_ alone.

**Figure 4:**
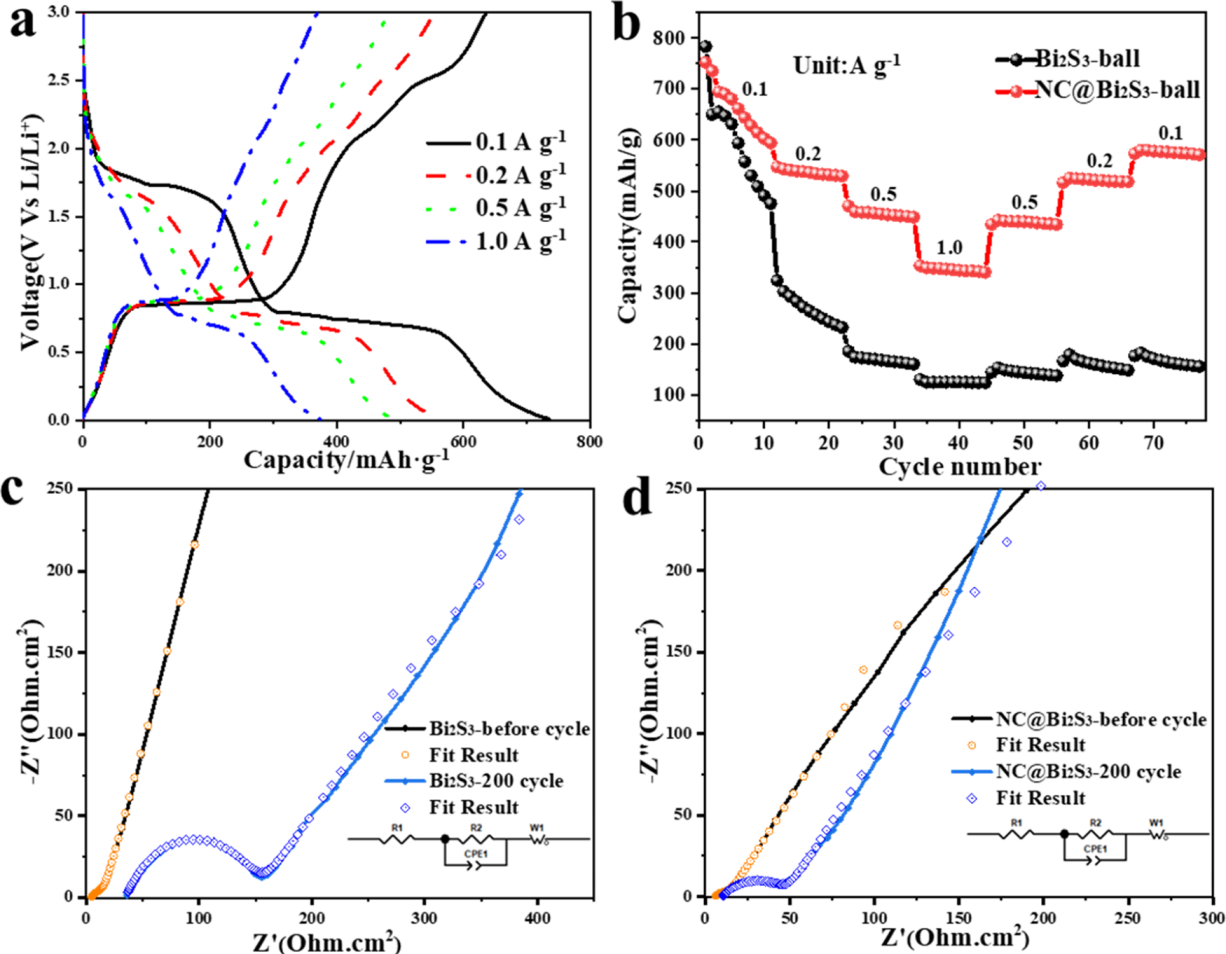
(a) Charge and discharge curves of NC@ Bi_2_S_3_ battery at current densities of 0.1, 0.2, 0.5, and 1.0 A/g; (b) Rate performance of Bi_2_S_3_ and NC@ Bi_2_S_3_ batteries at different current densities; (c) Impedance spectrum of Bi_2_S_3_ battery before and after cycling; (d) Impedance spectrum of NC@ Bi_2_S_3_ battery before and after cycling.

The rate performance of NC@Bi_2_S_3_ battery was tested, as shown in Figure 4a,b. Figure 4a shows the charge and discharge curves of NC@ Bi_2_S_3_ battery at different current densities of 0.1 A/g, 0.2 A/g, 0.5 A/g and 1.0 A/g, which corresponds well to the rate performance diagram of Bi_2_S_3_ and NC@ Bi_2_S_3_ batteries in Figure 4b. (The charge and discharge curves of the bare Bi_2_S_3_ cell at current densities of 0.1, 0.2, 0.5, and 1.0 A/g are shown in Figure S7). Among them, the uncoated Bi_2_S_3_ battery exhibits poor rate performance. After charging and discharging at a large current density, its structure is destroyed, and its performance cannot be restored when it is discharged back to a low current density. This shows that due to its limited conductivity and drastic volume changes during the lithiation/attenuation reaction, it cannot withstand fast discharge/charge rates. In contrast, the NC@Bi_2_S_3_ battery has a capacity of 643.91, 536.13, 454.72, and 345.32 mAh/g at current densities of 0.1, 0.2, 0.5, and 1 A/g, respectively. When the current density is restored to 0.1 A/g, the reversible capacity of NC@Bi_2_S_3_ gradually recovers to 576.31 mAh/g, which indicates that even in the process of rapid lithiation/decay, the active NC@Bi_2_S_3_ material is tightly fixed on the electrode, showing good rate performance. After high-rate charge and discharge, the battery performance is basically unchanged when it returns to low-rate charge and discharge, and can be restored to the performance of the first charge and discharge at this rate. The resistance characteristics of the Bi_2_S_3_ and NC@ Bi_2_S_3_ batteries are shown in Figure 4c,d. Table S1 shows the specific values of EIS. The oblique line at low frequency and the semicircle at medium and high frequency correspond to the Warburg diffusion impedance (R_1_) and charge transfer resistance (*R*_CT_), respectively. From the data, we can observe that the *R*_CT_ of the NC@Bi_2_S_3_ electrode (8.43 Ohm) is lower than that of the pure Bi_2_S_3_ electrode (11.09 Ohm), proving that the soft carbon coating on the Bi_2_S_3_ submicrospheres can promote the charge transfer reaction.^23^ After the Bi_2_S_3_ and NC@ Bi_2_S_3_ batteries were cycled for 200 cycles, the interfacial impedance value of the uncoated Bi_2_S_3_ battery increased greatly. This is because the volume expansion rate of the Bi_2_S_3_ material is large during the cycle, and the surface SEI layer is constantly broken and reformed, resulting in the formation of a thick SEI layer on the surface. The interfacial impedance value of the NC@ Bi_2_S_3_ battery only increased slightly, indicating that the nitrogen-rich carbon layer coating can play a good inhibitory role in volume expansion. However, its internal resistance increased from 5.78 to 10.12 Ω. It may be that the structure of the nitrogen-rich carbon layer was destroyed during the expansion and contraction of the material, resulting in a decrease in conductivity and an increase in internal resistance.

**Figure 5:**
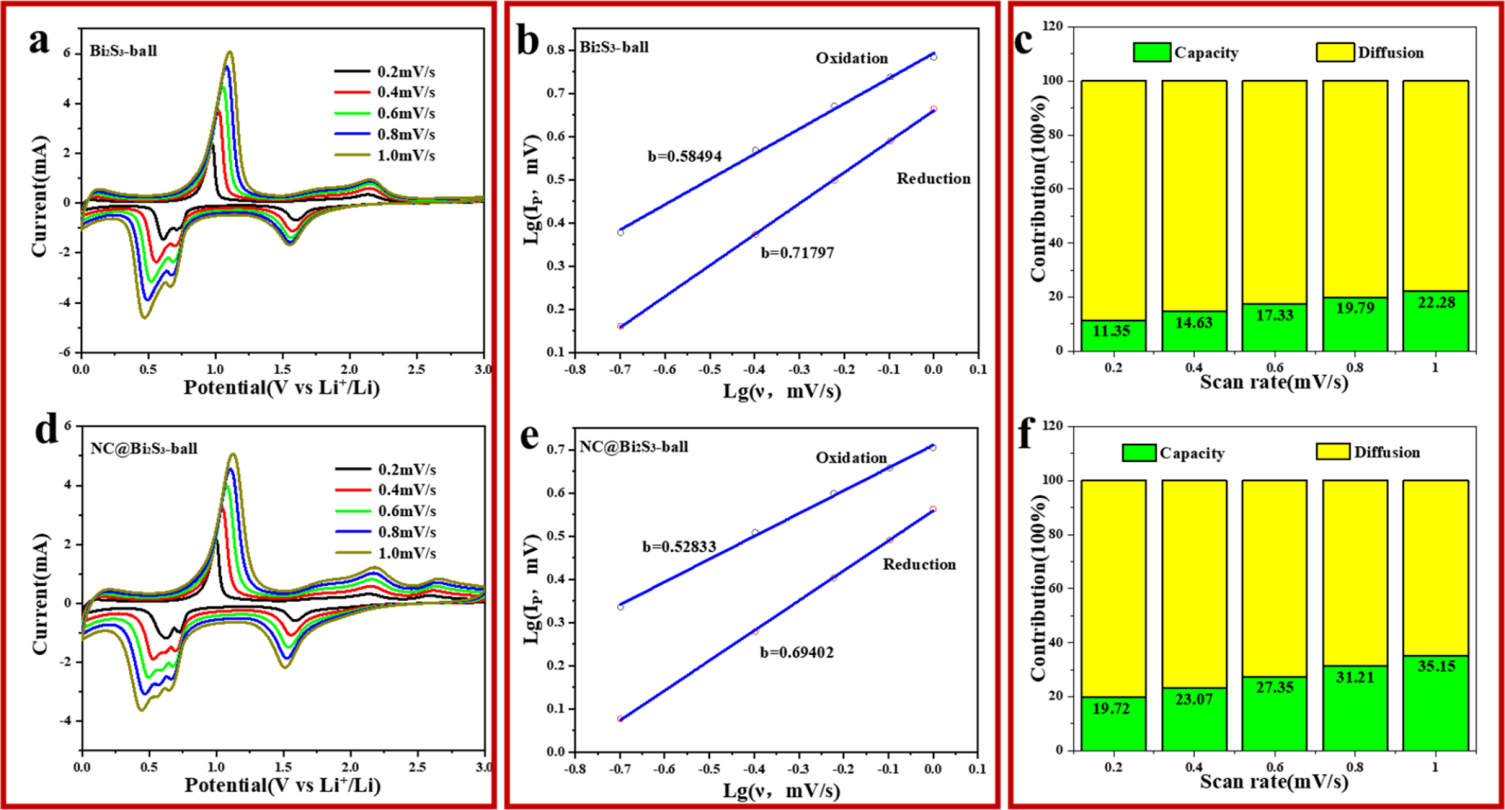
(a) CV curves of Bi_2_S_3_ battery at different scan rates (0.2–1.0 mV/s); (b) The functional relationship between the peak current value and the scan rate in the CV curve of Bi_2_S_3_ battery during Li alloying and dealloying; (c) The proportion of diffusion behavior and pseudocapacitive behavior during the charge and discharge process of Bi_2_S_3_ battery; (d) CV curves of NC@Bi_2_S_3_ battery at different scan rates (0.2–1.0 mV/s); (e) The functional relationship between the peak current value and the scan rate in the CV curve of NC@Bi_2_S_3_ battery during Li alloying and dealloying; (f) The proportion of diffusion behavior and pseudocapacitive behavior during the charge and discharge process of NC@Bi_2_S_3_ battery.

Next, we analyzed the kinetic performance of Bi_2_S_3_ and NC@ Bi_2_S_3_ batteries. The good electrochemical performance of NC@ Bi_2_S_3_ batteries is related to their reaction kinetics. We performed CV tests at different scan rates to explore their kinetic characteristics. The results are shown in Figure 5a,d.

The CV curves of Bi_2_S_3_ and NC@Bi_2_S_3_ batteries at different scan rates are highly similar and have no obvious distortion. As the scan rate increases, the peak position gradually shifts. This shows that before and after the nitrogen-rich carbon layer is coated, the Bi_2_S_3_ battery has a stable structure and can support highly reversible electrochemical reactions.^24^ We select the reduction peak current corresponding to the lithium alloying of two anodes and the oxidation peak current corresponding to the lithium dealloying, and calculate their functional relationship with the scan rate. The functional relationship can be obtained by the following formula:
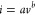
3
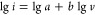
4Where *i* is the peak current value, *v* is the scan rate, and *a* and *b* are adjustable parameters.^25^ The peak current and scan rate are logarithmically fitted according to eq 4 to obtain the results shown in Figure 5b,e. The *b* value is the slope of the line. According to the size of the *b* value, we can determine the kinetic properties of the material. Generally, if the *b* value is close to 0.5, the electrochemical kinetic process is controlled by diffusion behavior. If the *b* value is close to 1, it is controlled by pseudocapacitive behavior. If the *b* value is between 0.5 and 1, the kinetic process is controlled by both diffusion behavior and pseudocapacitive behavior.^26^ The *b* values corresponding to the reduction peak and oxidation peak of the uncoated Bi_2_S_3_ battery are 0.58 and 0.72, respectively, indicating that the electrochemical kinetics of Bi_2_S_3_ is controlled by both diffusion and pseudocapacitance behaviors, and that it is not a pure battery material.^27^ When the Bi_2_S_3_ battery is coated with a nitrogen-rich carbon layer, the *b* value of its reduction peak remains almost unchanged, but the *b* value of the oxidation peak decreases. At this time, the contribution of pseudocapacitance to the battery’s kinetics increases.

According to the *b* value of the two Bi_2_S_3_ batteries between 0.5 and 1.0, we can accurately calculate the proportion of pseudocapacitance control and diffusion control according to the following formula:
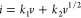
5
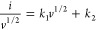
6

In the formula, *i* and *v* are still the peak current value and the scan rate, and *k*_1_ and *k*_2_ are constants.^28−30^ The results are shown in Figure 5c,f. As the scan rate increases, the pseudocapacitance ratio also increases. This is because pseudocapacitance is mainly generated by the fast Faraday process on the surface of the material.^29^ The faster the scan rate, the shorter the time for ions to diffuse and penetrate into the bulk phase, resulting in the inability of the material bulk phase to keep up with the diffusion-controlled kinetic process.^31−33^ Therefore, the diffusion control ratio decreases, and the contribution of pseudocapacitance increases. The pseudocapacitance ratio of the uncoated Bi_2_S_3_ battery is low, while the Bi_2_S_3_ battery coated with a nitrogen-rich carbon layer has a larger pseudocapacitance ratio, which may be because the coating of the nitrogen-rich carbon layer reduces the ion diffusion rate, so the surface Faraday behavior is more significant.^34^ By observing the cycle performance diagram in Figure 3d, it can be found that the cycle performance curve of the NC@Bi_2_S_3_ battery has a process of first decreasing and then increasing. This is because as the number of cycles increases, the pseudocapacitance will gradually disappear, so the capacity decreases, and the subsequent capacity increase may be the result of further release of the capacity of the bulk phase of the material.^35−37^

**Figure 6:**
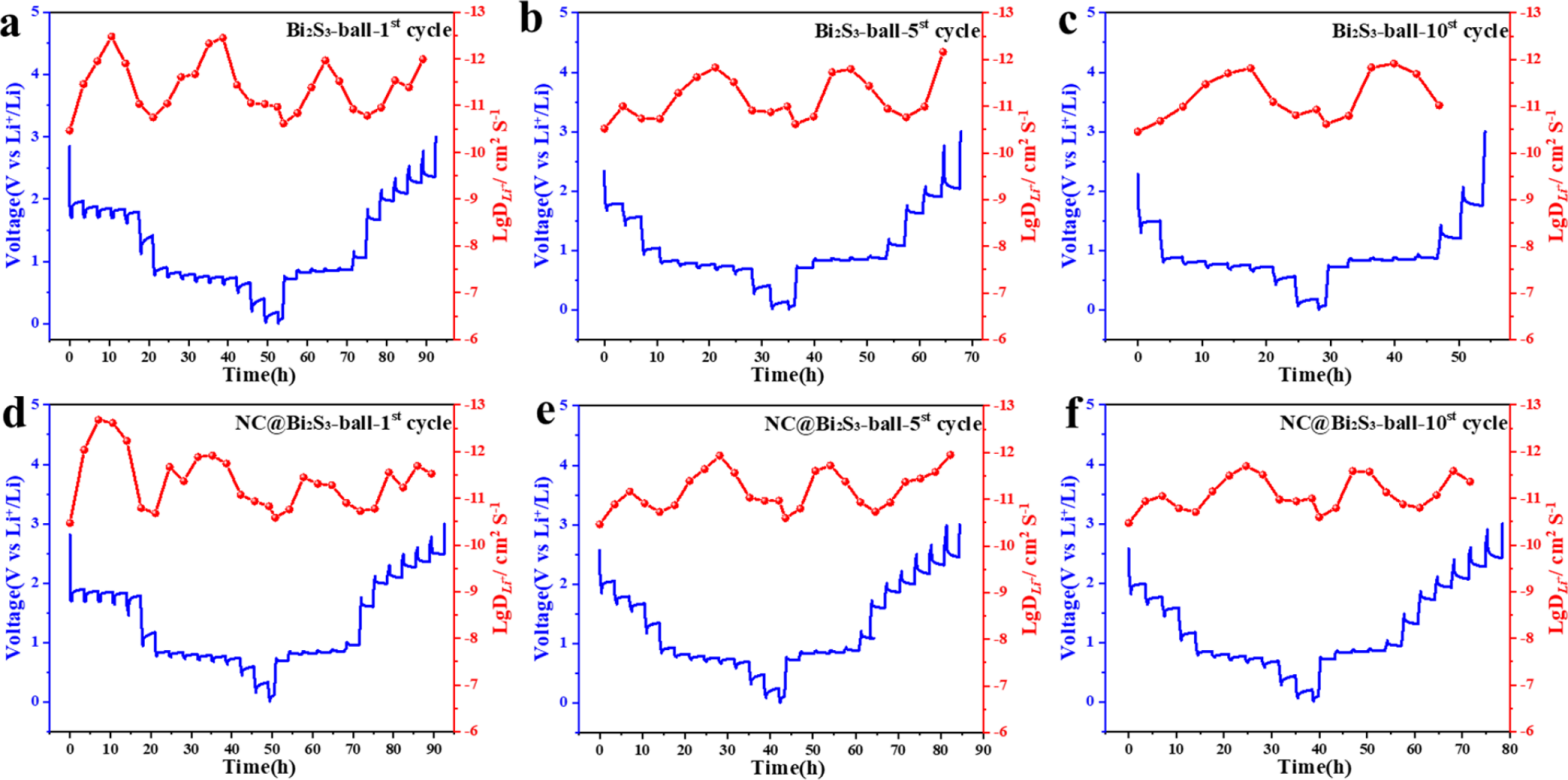
Diffusion coefficient and voltage distribution of Bi_2_S_3_ battery with different cycles of GITT test (a) 1 cycle; (b) 5 cycles; (c) 10 cycles; Diffusion coefficient and voltage distribution of NC@Bi_2_S_3_ battery with different cycles of GITT test (d) 1 cycle; (e) 5 cycles; (f) 10 cycles.

As shown in Figure 6, the distribution of the diffusion coefficients of Bi_2_S_3_ and NC@Bi_2_S_3_ batteries at different cycle numbers is characterized. It can be observed in the GITT diagram that with the formation of SEI, the diffusion coefficient of the material tends to be stable. It can be observed from Table S2 that the diffusion coefficient of the Bi_2_S_3_ battery continues to increase with the increase in the number of cycles. It may be that in the early cycles, the material continued to pulverize, forming multiphases, the number of multiphase interfaces increased, and the number of ion transmission channels formed also increased, so the diffusion coefficient continued to increase. In addition, the pulverization of the material leads to an increase in the specific surface area, and the increase in the contact area with the ions increases the number of ion embedding channels, which also leads to a larger diffusion coefficient.^38−40^ The nitrogen-rich carbon layer coating can reduce the pulverization of the material, so its diffusion coefficient value quickly tends to be stable, and the diffusion coefficient values of the fifth cycle and the 10th cycle are basically unchanged. At the same time, we can observe that after Bi_2_S_3_ is modified by nitrogen-rich carbon layer coating, its diffusion coefficient value is not much different from that of the uncoated Bi_2_S_3_ material. This may be because the coating effect of the nitrogen-rich carbon layer is uniform and does not cause obstruction of the ion transmission channel, because the diffusion coefficient has no obvious change.^41^

**Figure 7:**
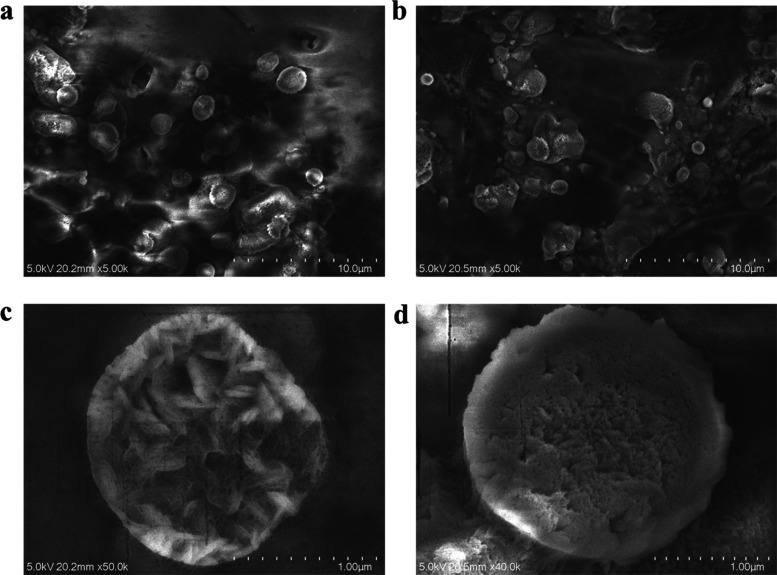
SEM images (a and b) of Bi_2_S_3_ and NC@Bi_2_S_3_ batteries after 200 cycles; high-resolution images (c and d).

The SEM images of Bi_2_S_3_ and NC@Bi_2_S_3_ batteries after 200 cycles were observed by scanning electron microscopy, as shown in Figure 7. Under high resolution, it can be clearly observed that the surface of Bi_2_S_3_ without nitrogen–carbon coating changes significantly during the cycle, resulting in crushing and collapse, and the structure is damaged; in contrast, the spherical structure of NC@Bi_2_S_3_ remains intact, proving that the nitrogen-rich carbon layer can inhibit volume expansion, so that its structure can still be well preserved after long-term cycling.^42^

## 4 Conclusions

In this paper, spherical Bi_2_S_3_ was synthesized by a simple hydrothermal method, and a nitrogen-rich carbon layer was successfully coated on its surface through a subsequent mild treatment. The lithium-ion battery anode NC@ Bi_2_S_3_ was successfully synthesized. The anode material was studied for microstructural characterization and electrochemical performance. Compared with the uncoated Bi_2_S_3_ battery, the NC@ Bi_2_S_3_ battery has better lithium storage performance; NC@ Bi_2_S_3_ still maintained the microspherical morphology, which could protect the internal Bi_2_S_3_ core from structural pulverization, while the surface of the uncoated Bi_2_S_3_ microspheres had been crushed. After 300 cycles at 100 mA/g, the specific capacities of Bi_2_S_3_ and NC@ Bi_2_S_3_ reached 340.99 and 645.13 mAh/g, respectively, indicating that the coating of the nitrogen-rich carbon layer can alleviate the problem of capacity fading. The ion diffusion rate weakens and the Faradaic behavior on the Bi_2_S_3_ surface becomes more significant after coating, resulting in a 10% increase in the contribution of pseudocapacitive behavior. The submicron spherical structure and simple synthesis process of NC@ Bi_2_S_3_ will be very suitable for practical electrode manufacturing technology of lithium-ion batteries, and also provide a strategy for the future development of bismuth-based alloy anode.

## Supporting Information Available

The samples were measured by XPS full spectrum; TGA measurement of thermal gravimetric analysis; high-resolution SEM characterization of sample morphology; low-resolution SEM characterization of sample distribution; BET measurement of sample specific surface area; electrochemical curves obtained by CV and charge–discharge cycles; and sample impedance and diffusion coefficient obtained by EIS and GITT tests (PDF)
