# Supplementary material for: NC@Bi2S3 Nanospheres as High-Performance Anode Materials for Lithium-Ion Batteries
Source: ACS Omega. 2024 Nov 26;9(49):48755–65. doi: 10.1021/acsomega.4c08339 (PMC11635501; doi:10.1021/acsomega.4c08339)
Supplement: Supplementary file 1 — ao4c08339_si_001.pdf [file ao4c08339_si_001.pdf]

Supporting Information

$NC@Bi_2S_3$ nanospheres as high-performance anode materials for lithium-ion batteries

*Wanda Kang[a), Sen Li[a), Xingchen Liu[a), Kun Yan[a), Wengao Zhang[a), Youkang Fan[a), Yuxiang Pan[a) and Jun Feng[a, b, *)*

[a] Department of Materials Science and Engineering, Southern University of Science and Technology, Shenzhen 518055, Guangdong, China.

[b] Guangdong Provincial Key Laboratory of Functional Oxide Materials and Devices, Southern University of Science and Technology, Shenzhen 518055 Guangdong, China.

[*] Corresponding author.
E-mail address: fengj@sustech.edu.cn

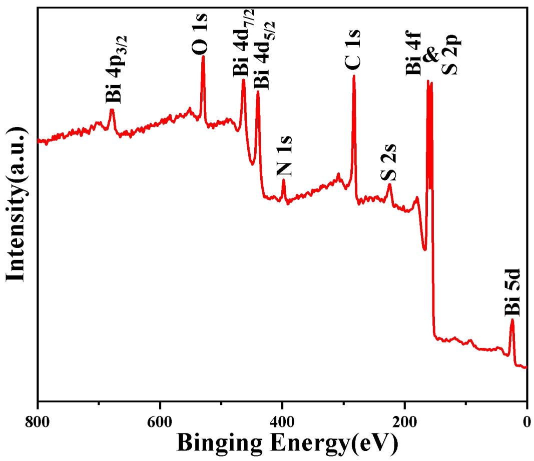

Figure S1 Full XPS spectra of NC@ Bi_2S_3 composites.

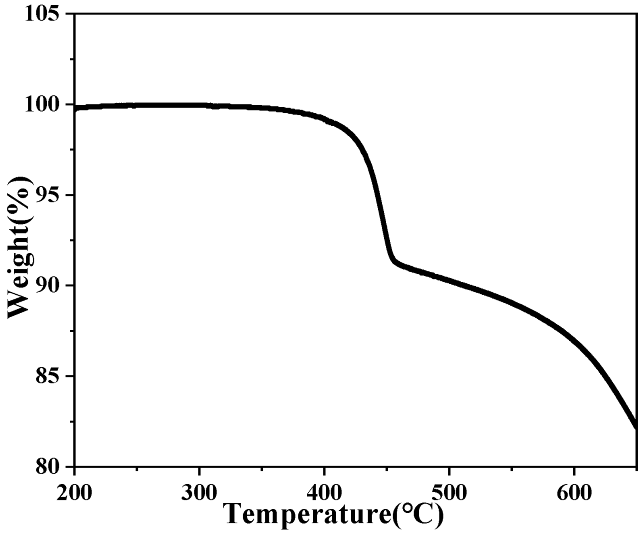

Figure S2 TGA image of NC@Bi_2S_3 composites material.

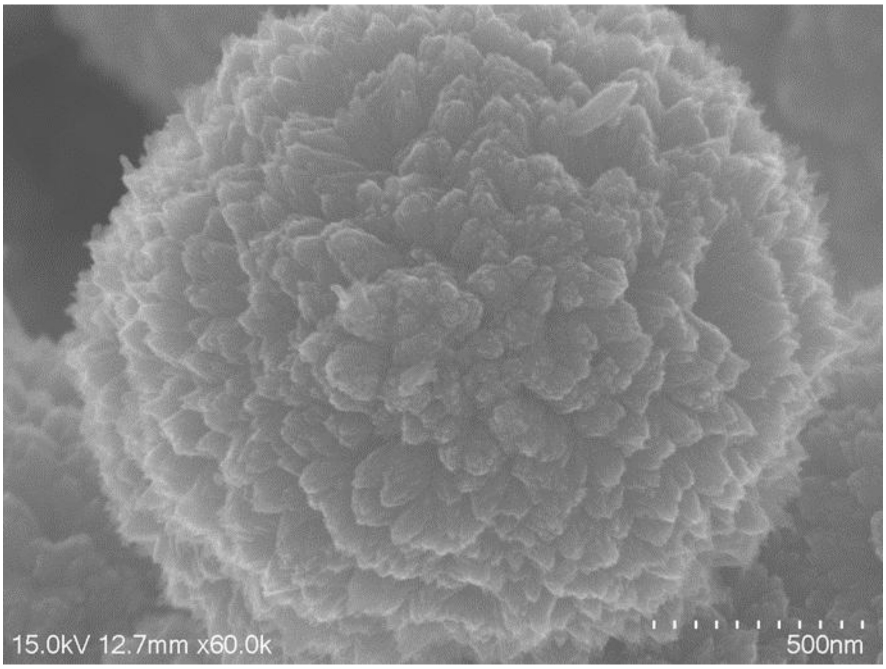

Figure S3 High-resolution SEM surface image of spherical Bi_2S_3.

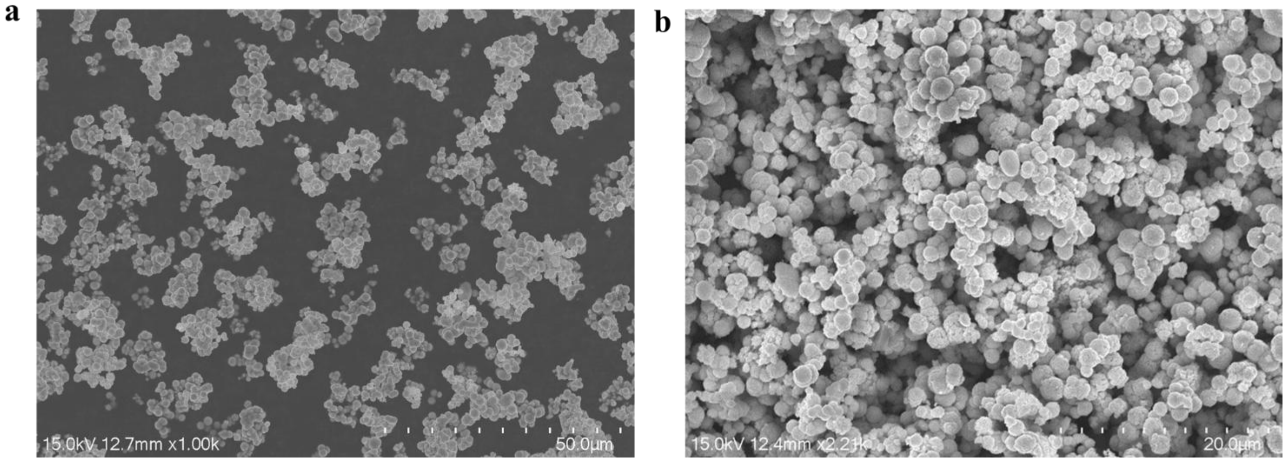

Figure S4 Low-resolution SEM images of (a)Bi_2S_3 and (b)NC@Bi_2S_3.

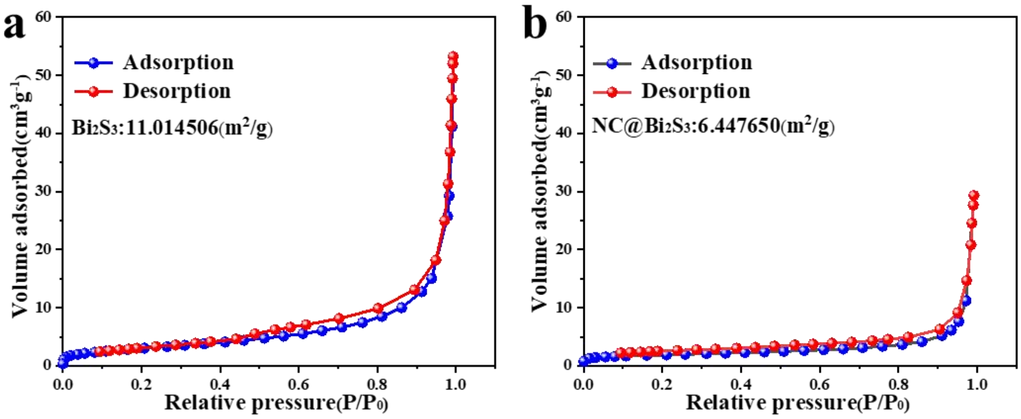

Figure S5 N_2 adsorption/desorption isotherms of (a) Bi_2S_3 and (b) NC @ Bi_2S_3 composites.

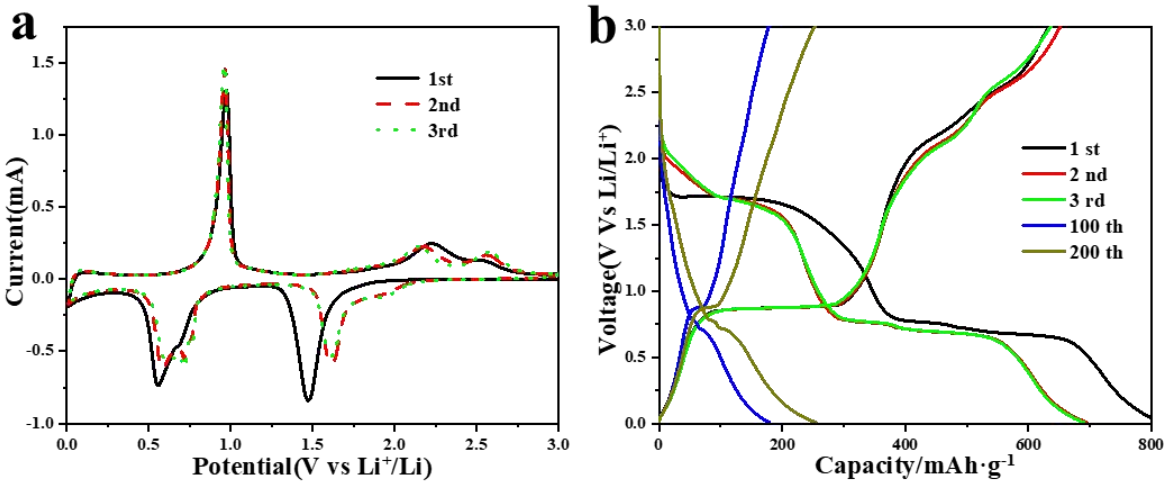

Figure S6 (a) Cyclic voltammetry curves of Bi_2S_3 battery at a scan rate of 0.1 mV/s for the first three charge and discharge cycles ;(b) Constant current charge and discharge curves of Bi_2S_3 battery at different cycle numbers at a current density of 0.1 A/g.

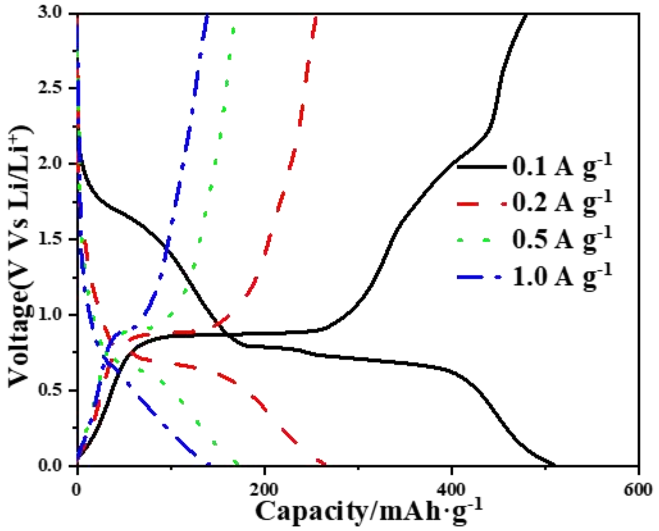

Figure S7 Charge and discharge curves of Bi_2S_3 battery at current densities of 0.1 A/g, 0.2 A/g, 0.5 A/g and 1.0 A/g.

Table S1 EIS data of Bi_2S_3 and NC@ Bi_2S_3 batteries before and after cycling.

**Sample**	**Cycle**	**R_1(Ω)**	**R_{CT}(Ω)**
**Bi_2S_3**	Before cycle	4.10	11.09
**Bi_2S_3**	After 200 cycle	35.18	116.30
**NC@Bi_2S_3**	Before cycle	5.78	8.43
**NC@Bi_2S_3**	After 200 cycle	10.12	38.00

Table S2 Diffusion coefficient values of Bi_2S_3 and NC@Bi_2S_3 batteries.

**Sample**	**Lg D ($cm^2 \cdot S^{-1}$)**		
	**1 Cycle**	**5 Cycle**	**10 Cycle**
**Bi_2S_3**	-11.3897	-11.1604	-11.1864
**NC@Bi_2S_3**	-11.3595	-11.1818	-11.0908
